# Surveillance for Creutzfeldt-Jakob disease in China from 2006 to 2007

**DOI:** 10.1186/1471-2458-8-360

**Published:** 2008-10-18

**Authors:** Qi Shi, Chen Gao, Wei Zhou, Bao-Yun Zhang, Jian-Ming Chen, Chan Tian, Hui-Ying Jiang, Jun Han, Ni-Juan Xiang, Xiao-Fang Wang, Yong-Jun Gao, Xiao-Ping Dong

**Affiliations:** 1 State Key Laboratory for Infectious Disease Prevention and Control, National Institute for Viral Disease Control and Prevention, Chinese Center for Disease Control and Prevention, 100 Ying-Xin Rd, Beijing 100052, PR China; 2 Chinese Center for Disease Control and Prevention, 27 Nan-Wei Rd, Beijing 100050, PR China

## Abstract

**Background:**

Human transmissible spongiform encephalopathies (HTSE), or Creutzfeldt-Jakob disease (CJD), is a group of rare and fatal diseases in central nervous system. Since outbreak of bovine spongiform encephalopathy (BSE) and variant CJD, a worldwide CJD surveillance network has been established under the proposition of WHO. In China, a national CJD surveillance system has started since 2002. The data of CJD surveillance from 2006 to 2007 was analyzed.

**Methods:**

Total 12 provinces are included in CJD surveillance system. The surveillance unit in each province consists of one or two sentinel hospitals and the provincial CDC. All suspected CJD cases reported from CJD surveillance were diagnosed and subtyped based on the diagnostic criteria for CJD issued by WHO.

**Results:**

Total 192 suspected CJD cases were reported and 5 genetic CJD, 51 probable and 30 possible sporadic CJD (sCJD) cases were diagnosed. The collected sCJD cases distribute sporadically without geographical clustering and seasonal relativity and the highest incidences in both probable and possible sCJD cases appeared in the group of 60–69 year. The most common three foremost symptoms were progressive dementia, cerebellum and mental-related symptoms. The probable sCJD patients owning both typical EEG alteration and CSF protein 14-3-3 positive have more characteristic clinical syndromes than the ones having only one positive. The polymorphisms of codon 129 of all tested reported cases shows typical patterns of Han Chinese as previous reports, that M129M are predominant whereas M129V are seldom.

**Conclusion:**

Chinese CJD patients possessed similar epidemiological and clinical characteristics as worldwide.

## Background

The description of human transmissible spongiform encephalopathies (HTSE) which includes a group of rare, fatal central nervous system disorders began with Creutzfeldt-Jakob disease (CJD), identified in the 1920s by two German neuroscientists[1]. Four types of CJD named sporadic (sCJD), familial or genetic (gCJD), iatrogenic or accidental (iCJD) and variant (vCJD) have been described. The pathological agent of this kind of diseases, termed as PrP^Sc^, is believed to be an abnormal isoform of a cellular prion protein, PrP^C^[2]. sCJD occurs spontaneously with unknown aetiology and has no evidence of geographical clustering. The incidence is generally between 0.5 and 1.5 cases per million persons per year and affects mainly the elderly population. Mutations in the gene encoding PrP^C ^(*PRNP*) is known as gCJD[3]. iCJD is caused by exposure to infectious prions through contaminated medical products such as biological materials and surgical instruments[4]. vCJD is caused by consumption of contaminated food through uptaking of bovine spongiform encephalopathy (BSE) prions[5].

Since the first ten vCJD cases were announced in the UK in March, 1996, the geographical association with BSE epidemic has been raised the possibility of a causal link. Up to April, 2008, total 204 vCJD cases were found worldwide, among them 166 cases were in UK including 115 cases died from definite vCJD, 48 cases died from probable vCJD without neuropathological confirmation and 3 are still alive [6]. In 2005, a vCJD case who had resided in the UK for 24 days was identified in Japan[7]. As the great impact of the outbreak of BSE and emerging of vCJD on public health, WHO consultation recommended the establishment of worldwide CJD surveillance in May, 1996. However, as the systematic surveillance for CJD has only been undertaken in a minority of countries, the incidence in much of the world area is currently unknown[8]. In China, the CJD surveillance system was established under the framework of the surveillances for communicable diseases led by Chinese Center for Disease Control and Prevention (CCDC) since 2002 and became broader in the past two years. The paper collects the surveillance data from 2006 to 2007.

## Methods

### Construction of CJD surveillance system

China CJD surveillance started in 2002, which was constructed under the framework of national surveillance network for communicable diseases led by CCDC. China has 31 provinces with a population of 1.3 billion. In CJD surveillance system, total 12 provinces with the population of 440 million were covered, including Beijing, Shanghai, Tianjin, Chongqing, Jilin, Shaanxi, Hubei, Guangdong, Guizhou, Anhui, Henan and Xinjiang. The surveillance unit in each province consists of one or two sentinel hospitals and the provincial CDC. The staff in the department of neurology in the sentinel hospitals was responsible for collecting the clinical data and sampling, while the staff from provincial CDC took charge in collecting the epidemiological data. The provincial CDC transferred all collected data and samples to the national center, which is the Department of Prion Disease, National Institute for Viral Disease Control and Prevention, CCDC, for laboratory tests and final diagnosis. The clinical and epidemiological data were collected by the formulized tables. The clinical data included mainly general information, main clinical manifestations, the first onset symptom, clinical examinations (CT, MRI, EEG and routine cerebral spinal fluid (CSF) biochemistry), specimen sampling data and death data. The epidemiological data included the information of inhabitancy, family history (mainly the dementia), anamnesis (surgical or neurosurgical history, organ transplantation, blood donation and transfusion, use of extracts of pituitary or other blood products) and special profession (medical staff, veterinary and butcher). The clinical specimens collected for diagnosis of CJD were brain tissues, CSF and blood. The national center feeds back the diagnosis and the principles of management to the provincial units. The surveillance and study was approved by the Ethical Review Committee of China CDC.

### Case definition

All suspected CJD cases reported from CJD surveillance were diagnosed and subtyped based on the diagnostic criteria issued by CCDC, which was constituted based on the diagnostic criteria for CJD issued by WHO[9]. The diagnosis for each case was made by an expert board consisting of neurologists, neuropathologists, epidemiologists and laboratory staff.

### Laboratory tests

For CSF 14-3-3 protein assay, CSF was collected by routine lumbar puncture during hospitalization. Western blots for protein 14-3-3 in CSF were performed as the protocol described elsewhere[10]. Briefly, 20 μl CSF sample was separated by 12% SDS-PAGE and electronically transformed onto nitrocellulose membrane. Blots were incubated in 1:1000 diluted 14-3-3 polyclonal antibodies (Santa Cruz, USA) and further incubated in 1:5000 diluted HRP-conjugated goat anti-Rabbit IgG (PerkinElmer, Germany). Immunoreactive bands were visualized by ECL method (PerkinElmer, Germany).

For *PRNP *analysis, genomic DNA was extracted from peripheral blood leukocytes by using Qiagen's DNA purification kit according to the manufacturer's instructions. The *PRNP *open reading frame was amplified by polymerase chain reaction (PCR) using a protocol and primers described elsewhere[11]. The genotype at codon 129 of *PRNP *was determined by digestion with the restriction endonuclease NspI. Analysis of *PRNP *sequences was performed by direct sequencing in a MacBAC sequencer (Pharmacia, USA).

For neuropathological assay, slides of brains were analyzed by HE staining. The immunohistochemistry staining for PrP^Sc ^were performed using PrP specific monoclonal antibody 3F4 (Dako) as the primary antibody. Western blots for PrP^Sc ^was conducted according to the protocol described previously[12]. Briefly, Brain tissue sample was homogenized in 9 volumes of lysis buffer (100 mM NaCl, 10 mM EDTA, 0.5% Nonidet P-40, 0.5% sodium deoxycholate, 10 mM Tris, pH 7.5). Aliquot of the brain homogenate was incubated with 50 μg/ml proteinase K (PK) at a final concentration at 37°C for 1 h. Samples were separated in 15% SDS-PAGE and electronically transferred to nitrocellulose membrane. The membrane was incubated with 1:5,000 diluted antibody 3F4 (Dako) and further incubated in 1:5000 diluted HRP-conjugated goat anti-Mouse IgG (Santa Cruz, USA).

### Statistic analysis

All statistical analyses were performed using the *SPSS *11.5 computer software programme.

## Results

From 2006 to 2007, total 192 suspected CJD cases were reported through CJD surveillance system. All reported cases were Han Chinese. After carefully analyzed the clinical, epidemiological and laboratory data, 51 patients were diagnosed as the probable sCJD cases, 30 were possible sCJD cases based on the diagnosis criteria. In addition, two fatal familial insomnia (FFI) cases in one family and 3 gCJD cases, G114V, T188K and E200K, were definitely diagnosed with the evidences of PrP^Sc ^in brain samples and/or special mutations in *PRNP*. Among them, two FFI cases and the G114V case had autopsy and were diagnosed as definite gCJD based on the neuropathological assays and Western blots. For every reported case, a well-regulated follow-up survey was carried out and registered individually, especially the patients with 14-3-3 protein positive in CSF. The final diagnoses of 29 non-CJD cases with positive 14-3-3 protein included 3 viral encephalitis, 1 syphilitic encephalitis, 2 tuberculosis encephalitis, 1 encephalomeningitis, 1 allergic encephalitis, 1 myelinoclasis, 2 neurodegenerative disease and 1 paraneoplastic syndrome. The rests discharged from hospitals without final definite diagnosis.

### General epidemiological data

**Figure 1:**
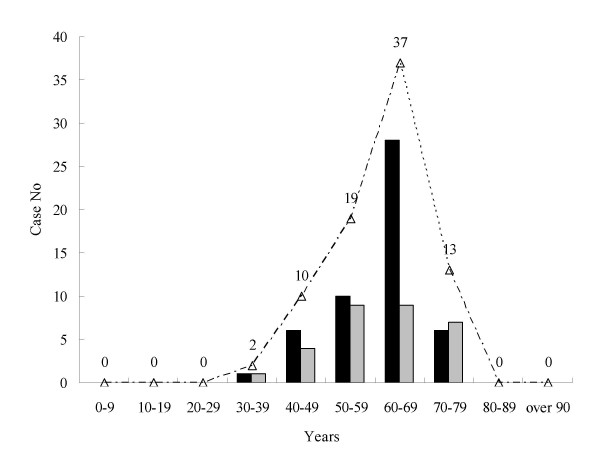
**The age distribution of probable and possible sCJD cases from 2006 to 2007.** The black column indicates probable sCJD, the grey column indicates possible sCJD and the curve represents the total numbers.

The onset ages of 51 probable sCJD cases varied from 33 to 74 years old, with the mean age of 60.2 years, and the onset ages of 30 possible sCJD were from 37 to 78 years old, with mean age of 60.4 years. The highest incidences were in the group of 60–69 year-old in both probable and possible sCJD cases (Fig 1). In contrast, the onset ages of 5 gCJD patients were from 26 to 63, with mean ages of 48.2 years. The male to female ratio was 35:16 in probable sCJD and 20:10 in possible sCJD.

The resident places of probable and possible sCJD cases were diffused in 23 different provinces in China, although Beijing and Shanghai reported more cases than other provinces. The occupations of probable and possible sCJD cases were also various, including workers, farmers, teachers, officials, self-employees and house-wives. Three cases were medical staffs before retired, but after careful investigation, we excluded the possibility of iatrogenic infection. All probable and possible cases showed no linkage with their anamnesis. Additionally, the suspected CJD cases were reported around all year, without seasonal specificity.

### Clinical data

The foremost symptoms of the reported sCJD cases were multifarious. Progressive dementia was the most familiar one that was described by 35 out of 81 (43.2%) patients. Other commonly observed clinical manifestations included mental-related syndromes (22/81, 27.2%), cerebellum-related syndromes (16/81, 19.6%), pyramidal or extrapyramidal disfunction (12/81, 14.8%), slow progressive dementia (8/81, 9.9%). Additionally, nine patients complained with visual disturbance, three with cerebral stroke-like symptoms without confirmation of ischemia, and rest three patients with dizziness, sleeping disturbance or lower limbs inability (Table 1).

**Table 1:** The foremost symptoms of the probable and possible sCJD patients

clinical manifestation	case number
progressive dementia	35 (43.2%)
mental syndrome^1^	22 (27.2%)
cerebellum syndrome	16 (19.8%)
pyramidal or extrapyramidal disfunction	12 (14.8%)
visual disturbance	9 (11.1%)
slow progressive dementia^2^	8 (9.9%)
cerebral stroke	3 (3.7%)
dizziness	1 (1.2%)
sleeping disturbance	1 (1.2%)
lower limbs inability	1 (1.2%)
clinical manifestation	case number

^1^: including sleeping turbulence, depression, anxiety and stress etc.

^2^: when the dementia persists longer than one year progressively without other manifestations

According to the diagnosis criteria, CJD must have progressive dementia and at least two of the following four clinical features, myoclonus, visual or cerebellar disturbance, pyramidal or extrapyramidal disfunction and akinetic mutism. Calculation of the presences of the four other manifestations among 81 sCJD cases identified that in the group of probable sCJD the most common symptom was pyramidal or extrapyramidal disfunction (84.3%), followed by myoclonus (78.4%), visual or cerebellar disturbance (68.6%) and akinetic mutism (47.1%), whereas in the group of possible sCJD the most common one was also pyramidal or extrapyramidal disfunction (86.7%), followed by myoclonus (70.0%), visual or cerebellar disturbance (70.0%) and akinetic mutism (50.0%) (Table 2). The appearance of common symptoms between probable and possible sCJD had no statistic difference, the p value showed in the table.

**Table 2:** The appearances of main clinical manifestations in probable and possible sCJD patients

	cases number	progressive dementia	myoclonus	visual or cerebellar disturbance	pyramidal or extrapyramidal disfunction	akinetic mutism
Probable sCJD	51	51 (100%)	40 (78.4%)	35 (68.6%)	43 (84.3%)	24 (47.1%)
Possible sCJD	30	30 (100%)	21(70.0%)	21(70.0%)	26(86.7%)	15(50.0%)
P			0.395	0.897	0.773	0.798

Analysis of the frequency of the four major manifestations in sCJD showed that besides progressive dementia, large portion of the patients had two other clinical symptoms, followed by having three and four (Table 3). The distributing frequency of the main clinical symptoms between probable and possible sCJD were quite similar, without statistic difference, the p value showed in the table.

**Table 3:** The frequencies of other four clinical manifestations except progressive dementia in probable and possible sCJD patients

	Case number	Having four clinical features	Having three clinical features	Having two clinical features
Probable sCJD	51	11 (21.6%)	17 (33.3%)	20 (45.1%)
Possible sCJD	30	7 (23.3%)	10 (33.3%)	16 (43.3%)
P		0.854	1.000	0.217

### Laboratory tests

Among 192 reported cases, 182 cases had the tests of protein 14-3-3 in CSF and 91 cases had EEG examinations. 65 CSF samples were 14-3-3 positive and 47 cases showed typical periodic sharp wave complexes (PSWC) on the EEG. Among 51 cases diagnosed as probable sCJD, 20 developed both CJD-specific EEG alternations and positive 14-3-3 in CSF, 16 were 14-3-3 positive only and 15 had EEG changes only. Comparative analyses of the emerges of the clinical manifestations showed that myoclonus was identified in all patients with both 14-3-3 positive and EEG change, while appearance frequencies of visual or cerebellar disturbance and akinetic mutism in this group were higher than that in the groups of 14-3-3 positive only and of EEG change only (Table 4). Most of the patients (93.8%) in the group of 14-3-3 positive only showed pyramidal or extrapyramidal disfunction, which was much higher than the other two groups. It highlights that the appearances of clinical manifestations may correlate with the phenotypes of laboratory examinations in sCJD patients. The patients with both 14-3-3 positive and EEG change seem to have more clinical symptoms. By statistic analyses, there was no difference in visual or cerebellar disturbance, pyramidal or extrapyramidal disfunction and akinetic mutism symptoms, but significant difference in myoclonus symptom among the three groups (p = 0.006, Table 4).

**Table 4:** Comparison of the results of 14-3-3 and EEG with the appearances of clinical manifestations in probable sCJD patients

	cases number	progressive dementia	myoclonus	visual or cerebellar disturbance	pyramidal or extrapyramidal disfunction	akinetic mutism
14-3-3 positive	16	16 (100%)	9 (56.3%)	9 (56.3%)	15 (93.8%)	6 (37.5%)
EEG changes	15	15 (100%)	11 (73.3%)	10 (66.7%)	13 (66.7%)	7 (46.7%)
14-3-3 + EEG	20	20 (100%)	20 (100%)	16 (80.0%)	15 (75.0%)	11 (55.0%)
p			0.006	0.306	0.293	0.579

Analyses of *PRNP *from 135 patients reported in CJD surveillance revealed again that most cases (131, 97.0%) were methionine homozygous genotype at codon 129, four (3.0%) were methionine/valine heterozygosity and none was valine/valine homozygous, which corresponding well with our previous results[13]. The polymorphism of codon 129 showed the same pattern in probable and possible sCJD patients, that in 48 tested probable cases 45 were M/M and 3 were M/V, and all 24 tested possible cases were M/M genotypes. The genotypes of codon 129 of three gCJD cases were all M/M.

## Discussion

Since German neurologists Creutzfeldt and Jakob firstly reported the cases with progressive cerebral dysfunction, the 'Creutzfeldt-Jakob disease' has been known for almost one century. Since the occurrence of vCJD in Europe was addressed, WHO consultation had recommended the establishment of worldwide CJD surveillance[14]. In 2002, China started to set up the national CJD surveillance and join into the worldwide surveillance system. It consists of twelve provinces now with about 440 millions inhabitants. In this paper, we propose the surveillance results from 2006 to 2007. Except five genetic CJD cases, all CJD cases are diagnosed as sCJD. No iCJD and vCJD has been identified. The collected sCJD cases distribute sporadically without geographical clustering. The mean onset ages of the probable sCJD and possible sCJD cases are almost the same, showing the highest incidence in the group of 60–69 years. There are more male cases than female ones in the past two years, probably reflecting only short-term surveillance.

Many symptoms have been described as the foremost clinical manifestations in sCJD patients. The first three common symptoms are progressive dementia, cerebellum and mental-related symptoms. Along with the progression of the disease, more neurological symptoms have been identified, among them progressive dementia has been observed sooner or later in all sCJD patients. No differences in the appearances and frequencies of other four main clinical manifestations have been notified in the groups of probable sCJD and possible sCJD.

EEG examination and CSF protein 14-3-3 test are the indexes for probable sCJD according to the diagnostic criteria recommended by WHO[9]. We find that the probable sCJD patients owning both typical EEG alteration and CSF protein 14-3-3 positive have more characteristic clinical syndromes than the ones having only one positive. Meanwhile, all cases with 14-3-3 positive and EEG altheration show myoclonus during their clinical courses. CSF 14-3-3 positive is believed as the marker of brain damage[15]. EEG abnormality represents early recognition of worsening brain function[16]. It might reflect that the patients owning two positive results have more pathological changes in brains.

In our surveillance system, the *PRNP *genes of all reported cases have been screened if available. The polymorphisms of codon 129 of all tested reported cases show typical patterns of Han Chinese as previous reports[13], that M129M are predominant whereas M129V are seldom. Additionally, analyses of a few biopsy and postmortem brain samples of sCJD cases collected previously show all type 1 PrP^Sc ^patterns in Western blots (unpublished data). All these patients have been confirmed to be M129M homozygous. Furthermore, five genetic CJD cases have been identified through CJD surveillance. Two cases, T188K and E200K, do not have detectable family histories, which are reported and primarily diagnosed as probable and possible sCJD, respectively. It emphasizes that *PRNP *gene sequencing is essential and unique for detection of gCJD without detectable family history.

Although CJD cases have been identified through our CJD surveillance, the reported and diagnosed cases is far from the expected one based on the numbers of inhabitants. Therefore, it is hard to line out the morbidity of CJD in China. Lack of knowledge of this rare disease in local clinicians in small town encumbers the sensitivity of surveillance system. Carefully designed training programs will help to improve this situation. Compared with the developed countries, the rate of postmortem is extremely lower in China, due to the traditional customs. Therefore, apart from the future legislation for brain autopsy of suspected CJD patient, enhancing follow-up will improve the quality of our CJD surveillance system.

## Conclusion

The results of the present study revealed the general epidemiology status of CJD in China from 2006 to 2007. Foremost clinical manifestations were different among the CJD patients, but along with the progression of the disease, progressive dementia was observed sooner or later in all cases. EEG examination, CSF protein 14-3-3 test and *PRNP *gene analyses were the main laboratory tools for the suspected patients without postmortem. M129M were the predominant genotype in Han Chinese. The morbidity of CJD in China still remained unsettled for the short-term surveillance. Due to the lower rate of postmortem, enhancing follow-up will improve the quality of the CJD surveillance system.

## Competing interests

The authors declare that they have no competing interests.

## Authors' contributions

QS and CG who were the principal investigators in the national CJD surveillance system contributed equally to this article. WZ performed Western blot for CSF 14-3-3 protein. B–YZ and J–MC performed the assays for PrPSc in brain tissues. CT and H–YJ performed *PRNP *gene analyses. JH, N–JX, X–FW and Y–JG were responsible for data collection and data analyses. X–PD was the group leader and the major contributor in writing the manuscript.

## Pre-publication history

The pre-publication history for this paper can be accessed here:


